# Inhibitory Learning during Exposure Treatment in Anorexia Nervosa: A Practical Guide

**DOI:** 10.3390/bs13050370

**Published:** 2023-04-29

**Authors:** Hanna Melles, Stefanie Duijvis, Anita Jansen

**Affiliations:** Department of Clinical Psychological Science, Faculty of Psychology and Neuroscience, Maastricht University, P.O. Box 616, 6200 MD Maastricht, The Netherlands; stefanie.duijvis@maastrichtuniversity.nl (S.D.); a.jansen@maastrichtuniversity.nl (A.J.)

**Keywords:** exposure therapy, inhibitory learning, anorexia nervosa, anxiety, avoidance, safety behaviors

## Abstract

Exposure therapy is known to be an effective intervention in the treatment of anxiety-related disorders. In eating disorders, such as anorexia nervosa, anxiety and avoidance are identified as maintenance factors. Therefore, they may constitute an important treatment target, suitable for the use of exposure therapy. Remarkably, exposure techniques to target fears and avoidance behaviors are not commonly used in the treatment of anorexia nervosa. We present a practical guide for the implementation of exposure therapy in the treatment of anorexia nervosa. We outline how exposure therapy is supposed to work according to the inhibitory learning model and how the exposure intervention can be designed for individuals with anorexia nervosa. Practical examples are provided through the case presentation of a patient with anorexia nervosa who completed 31 exposure sessions that focused on her fears of food, eating, weight, weight gain, their feared social consequences and the associated safety behaviors.

## 1. Introduction

Anorexia nervosa is a severe and difficult-to-treat eating disorder. Affected individuals are preoccupied with an obsessive drive towards thinness that can have devastating consequences, reflected in a mortality rate of ~5% [1,2]. About 30% of the patients do not respond to their treatments and remain chronically ill, resulting in illness durations of up to 20 years [3]. A recent meta-analysis showed that current psychological treatments for anorexia nervosa do not outperform the only moderately effective control conditions in terms of weight gain, eating disorder psychopathology and quality of life [4]. Anorexia nervosa is not only associated with a high disease burden; eating disorders also have a tremendous socioeconomic impact that exceeds USD 64 billion per year in the United States [5]. These findings are alarming and underline the need for improved treatments for eating disorders, such as anorexia nervosa.

Ideally, psychological treatments should target disorder-maintaining factors. Anxiety and avoidance have been identified as such in anorexia nervosa [6,7,8,9,10,11,12]. Patients with anorexia nervosa display a variety of anxieties; in addition to a strong fear of weight gain, they are afraid of food, eating and its consequences on a personal, physical and social level [6,9,11,13,14,15,16,17]. Fear learning processes are supposed to be involved in the manifestation of anxiety in eating disorders [7,9,10,16,18,19,20,21,22]. Pavlovian learning occurs when an initially neutral stimulus such as food (CS; conditional stimulus) becomes associated with an aversive or feared stimulus, such as weight gain (US; unconditional stimulus), and in this way becomes a predictor of threat [23,24]. The mere presence of food (CS) is then sufficient to activate fear memories and fear reactions (CR; conditional response [24]). The fear typically elicits avoidance behaviors, such as food restriction, to prevent the expected aversive outcomes [18,20,25,26,27,28,29,30,31]. The individual may expect the weight gain to be endless and out of control, and may learn to downregulate the anxiety by avoiding certain foods to prevent the expected uncontrollable weight gain [32]. While avoidance leads to an immediate reduction in anxiety, avoidance of the expected catastrophic outcome or its predictors withholds the opportunity to question the accuracy of the expectation. Restrictive eating behaviors prevent the patient from learning whether the expectations are correct, that is, whether eating high-caloric foods will actually lead to endless and uncontrollable weight gain. In consequence, avoidance behaviors are negatively reinforced, and anxiety is maintained or even increased in the long term [9,24,32,33,34,35,36,37,38,39].

An intervention that specifically targets anxiety and avoidance is exposure therapy, known as the gold standard treatment for anxiety-related disorders [40,41]. In exposure therapy, stimulus–outcome associations are challenged through extinction learning, a process that refers to learning from the absence of reinforcement, thereby reducing fear and fear reactions. This can occur by presenting the CS (e.g. food) in the absence of the US (e.g. uncontrollable weight gain) while preventing the use of avoidance behaviors [23,34,40,42]. Given the eminent role of fear and avoidance in anorexia nervosa, exposure therapy is a suitable candidate for its treatment [8,10,16,21,25,43,44,45,46]. Though the idea of using exposure therapy in anorexia nervosa is not new and several studies already indicate its effectiveness in reducing anorectic fears, the intervention is not yet systematically used in clinical practice [10,18,26,47,48,49,50,51,52]. The main reasons for the underuse of exposure therapy in clinical practice are clinician anxiety, concerns and negative attitudes towards the treatment (see, e.g., [53,54,55,56,57]).

Targeting anorectic fears using exposure therapy, however, can be particularly helpful in the context of weight restoration. While weight gain is a primary and inevitable goal of most anorexia nervosa treatments, it is also one of the patients’ most central fears and, therefore, highly anxiogenic. In fact, practitioners often believe that they use exposure interventions because patients need to eat and gain weight anyway. It is, however, a common misconception that the mere confrontation with food and eating during weight restoration programs is a form of exposure that is sufficient to address anorexia nervosa-related fears [58]. To achieve the desired change in fear memories and feelings of anxiety, it is necessary to set up exposure interventions very well. In this article, we discuss why this is the case and how it can be achieved. Hitherto, only a few clinical protocols on inhibitory learning-based exposure therapy in eating disorders exist (e.g., [58,59]), and structured guidance for the treatment of anorectic fears with exposure therapy is still scarce. First, inhibitory learning is introduced, followed by the application of inhibitory learning-based exposure therapy to anorexia nervosa, using a case description of a patient who completed 31 exposure therapy sessions.

## 2. Inhibitory Learning

In most previous studies of exposure therapy for anorexia nervosa, the intervention is built upon the habituation model of extinction (e.g., [10,26,48,49,50,60]). According to this model, weakening of the original CS-US association through exposure and response prevention and the consecutive habituation of anxiety during a therapeutic session is the assumed working mechanism of the therapy [10,61,62]. Anxiety levels at the end of a session, however, are not predictive of long-term treatment outcomes, suggesting that other processes such as inhibitory learning may be involved [63]. The inhibitory learning model states that in addition to the mere *exposure* to a feared stimulus, the disconfirmation of associated threat expectancies is a necessary component of exposure therapy [23,63,64]. Specific hypotheses about the often irrational threat expectations should be formulated and tested to build new, safe associations between stimulus and outcome. During the exposure, patients, for example, test and disconfirm the feared expectation that eating a cheese sandwich every day of the week (CS) will lead to endless weight gain (US). So far, however, only a single study by Cardi et al. [18] has investigated the isolated effects of inhibitory learning-based exposure therapy in anorexia nervosa. Though this innovative study examined a small sample, had no control condition, and mainly targeted fears of food while omitting other anorectic fears, the results are promising: the exposure was generally very well accepted and the BMI of the patients increased, while fears and avoidance behaviors decreased [18]. According to the inhibitory learning model, extinction learning involves the formation of a new (safer) memory in addition to the old (unsafe) memory, and with frequent exposures, the new safe memory becomes more dominant than the old (unsafe) memory. Therefore, the CS predicts two possible outcomes (US or no-US) after extinction [65,66]. The co-existence of two memories makes one vulnerable to spontaneous recovery of the original CS-US association [23,63,67]. The likelihood of this increases with the time that has passed between the last CS–no-US pairing [67], when the context changes [65,68], when initial CS-US pairings reoccur [69,70] or when unsignaled US presentations occur after extinction [71]. To reduce the chances of spontaneous reinstatement, several strategies to enhance inhibitory learning are advised [23,63,72]: *Expectancy violation*: exposure sessions should be built upon the premise that expectations are maximally violated; the bigger the mismatch between what the individual expects and what actually happens and the more ‘*surprising*’ the outcome, the stronger the inhibitory learning effect [73,74]. In this context, one should refrain from using cognitive strategies that are known to reduce anxiety levels prior to exposure sessions. Self-protective cognitions that reduce probabilities, such as ‘*I know nothing will happen*’—which, in our experience, are frequently used by patients with anorexia nervosa—can negatively impact the effect of an exposure since they reduce the mismatch between expectation and outcome [23,63]. *Deepened extinction* entails the combination of either previously extinguished stimuli or new stimuli that predict the same US after isolated extinction [75,76]. During *occasional reinforced extinction*, the initial CS-US association is sometimes presented again [77]. Similar to what is known from previously tested exposure treatments, in inhibitory learning-based exposure therapy, the use of *safety behaviors* should also be reduced. Throughout the therapy, the removal of safety signals/behaviors that the patient usually applies, such as purging behaviors, is desirable when they hinder/exacerbate the disconfirmation of threat expectancies. The use of safety behaviors may be allowed when they do not interfere with the violation of expectancies, such as slower eating, and when the patient would otherwise not complete the exposure at all [32,78]. Another strategy to enhance inhibitory learning involves *variability* in stimuli, contexts, timing, intensity and fear levels [23,63,79,80,81,82,83,84]. It is also important that the patient stays focused and keeps paying *attention to the CS,* to become aware of the new CS–no-US association [63]. These strategies can be complemented by *affect labeling* during exposure sessions and *mental rehearsal* of what was learned. This fosters inhibitory learning through linguistic processing and *memory consolidation* [23,79,85]. Below, it will be demonstrated how inhibitory learning-based exposure therapy can be used in the treatment of fears in patients with anorexia nervosa.

## 3. Before the Start of Exposure Therapy

In general, exposure therapy can be applied as a standalone treatment or in conjunction with other evidence-based interventions. In either case, the exposure sessions should be scheduled regularly. In order to build new stimulus–outcome associations (CS-no-US) that are strong enough to overrule the old ones (CS-US) in daily life, frequent practicing is necessary. The fear cognitions have often persisted for many years, leading to automated behaviors in patients. Furthermore, the implementation of the recommended strategies to enhance inhibitory learning and the internalization of the new learning take time and require repetition [63]. There is no fixed session length; a session should take as long as necessary to test and disconfirm the threat expectancy. Further, exposure sessions should vary in their level of difficulty. While in traditional forms of exposure therapy, gradually following a fear hierarchy is common, inhibitory learning benefits from varying the level of difficulty between and during the sessions [23]. This variation enables continuously high levels of anxiety instead of promoting habituation, which can be beneficial [23,79].

Before the actual exposure therapy is started, the patient should be familiarized with the rationale behind the treatment. Because the prospect of facing fears and stimuli that are usually avoided can in itself cause anxiety, it is important that the patient understands why the exposure is carried out and how it works [58,59]. An example of how to present and discuss the rationale for exposure treatment is provided in Appendix A. In addition to understanding the treatment rationale well, therapy motivation should continuously be addressed, as exposure requires a lot of courage (of both the patient and the therapist; see [58,59]). Patients should not be forced into exposure therapy but rather be guided to experience that their fears about, e.g., infinite and uncontrollable weight gain may prove not to be true. Performing exposure exercises and finding out that anxious prospects do not come true may be the most straightforward way to increase motivation. Therefore, also to boost motivation, the patient must do the therapy work, i.e., exposures to feared foods or weights, challenging fear expectancies and refraining from safety behaviors that prevent new learning.

It is also important to fully understand the patients’ fears [59]. To maximize the success of an exposure session, the correct CS must be chosen (identical CSs may have different meanings for different individuals) [16]. While one patient, for instance, fears that eating a sandwich causes immediate and never-ending weight gain, another patient associates the same CS with the fear of being judged by others for their food choice and/or the way they eat the food. It is advised to continuously collect the necessary information about (a) the anxiety-provoking cues and/or situations (CS), (b) the expected aversive outcome(s) (US), (c) how likely an expected outcome is for the patient and (d) whether and what safety/avoidance behaviors are used to prevent the outcome(s) [63].

## 4. Exposure Sessions

In the following section, an example will be presented on how exposure therapy can be applied to the fear of weight gain in anorexia nervosa, based on inhibitory learning guidelines [23,63]. An exposure form that can be used during the sessions, based on the work of [23,63,86] is provided in Appendix B. A patient, for instance, mentions that she is afraid of gaining weight (US) after eating sweets, such as chocolate (CS). In order to design a proper exposure exercise, the therapist collects detailed information about the CS: does the type of chocolate matter? The time of the day it is eaten? The rest of the meals planned that day? What amount of chocolate is expected to cause weight gain? In addition, detailed information about the US is needed, such as how much weight is expected to be gained? When will the weight gain be detectable (e.g. immediately after eating or on another day)? Once sufficient information about CS and US has been gathered, the individual’s expectation is formulated as a testable hypothesis. The hypothesis should enable the best possible test of the expectation and generate the biggest mismatch between expectation and outcome, e.g., ‘When I eat two pieces of milk chocolate (10 g) as an extra snack today, I will gain 0.5 kg by tomorrow’. Before conducting the actual exposure, the therapist also collects information about any safety behaviors that the patient may use to prevent the feared outcome. If the patient usually compensates for extra calories by skipping other meals or doing sports, it is important that she does not apply these behaviors during the exposure experiment, as such behaviors would sabotage the outcome. For example, if the patient starts exercising excessively after eating the chocolate, the isolated impact of eating chocolate on her weight cannot be determined. The relevance of refraining from compensatory (safety) behaviors when testing specific threat hypotheses should, therefore, be made explicit and clear to the patient before the actual exposure begins.

The chocolate exposure could then proceed as follows. The patient first rates how afraid she is and how strongly she believes that the expected outcome will occur. This can be achieved using visual analogue scales between 0 and 100, where 0 means that the patient is not afraid respectively that the aversive outcome will not occur and 100 means that the patient is very afraid respectively that the aversive outcome will definitely occur (see Appendix C). Next, the patient is weighed, in order to know her weight before eating the chocolate. The patient should be encouraged to look at the scale herself and to mention her weight. She is then asked to eat the two pieces of chocolate. During the exposure, the patient rates her anxiety again. Further, the therapist should take care that the patient does not use any safety behaviors that diminish the mismatch between expectation and outcome but ensure that she stays focused on the CS. Safety behaviors such as eating at a specific pace or in a certain way, or knowing the exact calories of the chocolate would be unrelated to the threat of gaining weight and, therefore, not impact expectancy violation, whereas eating less of the chocolate or purging afterwards would prevent full fear confrontation and, in consequence, hinder inhibitory learning.

One day after the exposure, the patient is weighed again. It is important that the patient is weighed on the same scale, in the same clothes and around the same time as at the first weighing to take natural weight fluctuations and other circumstances into account as much as possible. Afterwards, there should be enough time for a profound post-discussion of the exposure and its results. The two weights are compared, and it is asked whether the expected outcome actually occurred (yes/no) and whether the result is surprising. The threat expectation is rated again, as is fear. Then, therapist and patient discuss what the outcome means to the patient, how she feels, whether the exposure was tolerable and whether she managed to not apply any compensatory behaviors that might have influenced the result. The therapist stimulates mental rehearsal by asking the patient to formulate what she learned from this exposure. Anxiety is measured again, and how strong the threat expectation still is after taking into account the new information obtained from the exposure is rated. Therapist and patient also discuss how difficult the exposure was and what the next exposure might look like. In general, the therapist can derive the level of difficulty either from the anxiety and expectancy ratings made immediately before the actual exposure and/or by discussing it with the patient when planning the next exposure. This patient, for instance, learned that she did not gain 0.5 kg in one day after eating two pieces of chocolate. This elicited new fears such as ‘then I will gain the 0.5 kg tomorrow’ or ‘I am sure I will gain 0.5 kg after I eattwo pieces on two days’ or ‘I will gain 0.5 kg when I drink a hot chocolate with it’. These new fears and expectations are challenged in new exposure sessions. In case the patient did not manage to refrain from compensatory behaviors or to perform the whole exposure experiment, the reasons why are discussed and the exposure is re-designed in a doable manner. 

It may, of course, also happen that a patient actually gains some weight after a food exposure or even that she gains the feared amount of weight. Ultimately, of course, weight gain is one of the goals of any anorexia nervosa treatment. One could argue that the fear of weight gain is justified if eating leads to weight gain. However, being afraid to gain weight while being extremely underweight is rather irrational. The frightening thing for many patients is the fear that the weight gain will never stop. Therefore, we propose finding and testing expectations about irrational weight gain expectations, such as infinite weight gain, lots of kilos at once or unstoppable weight gain. It is rather unlikely that these expectations will materialize. Of course, even a small increase in weight will be very frightening for many patients, but then the question should be what exactly is so frightening about it. What is so bad about a 0.5 kg weight gain, knowing that you have to move towards a BMI of 18.5? Patients may then find out that they can tolerate more than previously expected, or there will be new feared expectations to test. Factual weight gain may be particularly frightening when it triggers fears of infinite or uncontrollable weight gain, becoming obese, losing control over intake forever and so on. These expectations can fuel new exposures, using weight gain as a CS to test new expectations, such as ‘when I gain 0.5 kg, I will continue to gain 0.5 kg every day’, ‘when I gain 1 kg, I will lose control over my entire life’ or ‘when I gain 1 kg, others think I am fat’. Exposure should teach patients that weight gain is neither infinite nor uncontrollable.

## 5. Case Description

The following section contains a case description of a patient with anorexia nervosa (Suzanna) who was treated with exposure therapy. She had previously undergone several other treatments for her eating disorder but still was very anxious (see Table 1). Over a four-month period, she completed two or three exposure sessions per week, with a total of 31 exposure sessions. A session lasted 30–60 min, depending on the specific exposure exercise performed. Most (*n* = 24) exposure sessions focused on food, eating and weight gain. The other seven sessions were scale exposures, that focused exclusively on her body weight.

Suzanna is a 22-year-old girl with a history of anorexia nervosa for about ten years. In her early adolescence, she was a member of a competitive sports club. Part of the training routine was regular weight measures before exercising. When the weight was not in a specific range, the athletes were punished. This weighing routine put a lot of pressure on the athletes—and too much pressure on Suzanna. She developed an increasing fear of being weighed in front of the team. Ultimately, she became afraid of being weighed in general, of others seeing and possibly commenting on her weight, of gaining weight and being too ‘fat’. She began to associate ‘weight gain’ with feelings of worthlessness, weakness, failure and loss of control, fueled by the underlying fear of being judged. Over time, she began to avoid weighing moments as much as possible, and when she had to be weighed, she avoided eye contact with the person weighing her, she obsessively weighed herself at home to prevent ‘surprise’ effects, wore light clothes only and restricted intake the day before being weighed. Eventually she started to restrict intake altogether which brought her to a low BMI of 15.2 several years ago. Several types of food she had previously enjoyed were now ‘forbidden foods’. These included, for instance, high-caloric foods such as fries. She developed fears related to eating and especially the social aspects: she was afraid of being the only one eating, of eating calorie-dense foods when others see that and of eating more than the others. Suzanna assumed she would gain more weight and faster than others, that others would comment on what and how she eats and that they consider her ‘fat’. Suzanna developed several strategies to regulate her fears about food and eating. She always waits until someone else starts eating and tries to make sure the other person eats as much or more, she eats very slowly to counter the fear of being judged as a ‘glutton’ and she talks a lot to distract herself and others from the fact she is eating. Suzanna suffers a lot from being constantly preoccupied with making decisions about food, performing compensatory behaviors and being torn between the desire and need for social contact and the huge fear of eating and being judged.

## 6. Practical Examples

Suzanna suffers from fears related to food, eating and weight gain, as well as their social consequences. Exposure therapy targeting these fears generates new learning, for example, about the relation between eating different foods and body weight. The exposures focused on the following goals: to reduce the list of forbidden foods; to find out that weight gain is neither uncontrollable nor never-ending, thereby stimulating feelings of being in control of her weight; to experience that she can tolerate eventual weight gain better than previously expected and to put the social relevance of food, eating and weight gain into a new perspective.

### 6.1. Fear of Food and Weight Gain

Suzanna experienced an intense fear of gaining weight very fast after consuming high-caloric or forbidden foods and liquids. As part of Suzanna’s exposure therapy, she ate and drank different types (such as breakfast, lunch, snacks, soft drinks, coffee, etc.) and amounts of foods at various times of a day (variability) in the therapeutic setting, at home or other places (different contexts). Typical hypotheses that were tested with Suzanna were, for instance, ‘If I eat my planned meals today, then I will weigh 0.5 kg more tomorrow’, ‘If I drink a hot chocolate, I will gain 0.5 kg directly’. The challenge with exposures entailing food consumption followed by immediate weighing is that the scale may actually display a slightly higher weight. The therapist should prepare the patient for this and emphasize that this does not represent actual weight gain but just the factual weight of the food that has been eaten before, spontaneous fluctuations or measurement errors. Such exposures can build the starting point for additional exposures. Even though Suzanna recognized that drinking a hot chocolate would not result in an immediate gain of 0.5 kg, the scale indicated a 0.2 kg increase in weight (weight of the drink) and she was afraid that the 0.2 kg would remain and even increase the next day, leading to a new hypothesis that could be investigated: ‘When I drink a hot chocolate, I will gain 0.2 kg every next day’, and so on. Typical conclusions that Suzanna drew from such food exposures were, for instance, ‘If I drink a hot chocolate, I will not immediately gain 0.5 kg’ or ‘I will not gain 0.3 kg immediately after eating a granola bar but only the actual weight of the food, which is 40 g, and this is not detectable anymore on the next day’.

Alongside the fear of eating specific foods, Suzanna was also afraid of eating spontaneously and of eating without knowing the exact number of calories that the food contained. She expected ‘If I eat a portion of chips without knowing how many calories it contains, I will immediately gain 1 kg’. To challenge this fear, the therapist can surprise the patient with a food exposure. An advantage of unexpected exposures is less likelihood of preparatory compensatory behaviors (e.g., food restriction, exercising, looking up calories) before the session. After the unexpected exposure, Suzanna concluded: ‘When I eat a portion of chips without knowing the number of calories, I don’t gain 1 kg immediately but maybe within a day’, which invited a test of the next expectation. When the patient fears never-ending weight gain after eating a specific food, the weight could be measured repeatedly over a longer period of time. Additionally, multiple types of measures could be used; some patients prefer to use measuring tapes to check the circumferences of arms, legs or belly, for instance, when they expect to detect weight gain. 

Patients with anorexia nervosa may fear that eating calorie-dense food once causes strong cravings, leading to bingeing behaviors that will never stop and result in weight gain. Suzanna expected that eating a portion of fries for lunch would make her eat unhealthily all day. Other common expectations of Suzanna were ‘If I eat two croissants, I will exchange all remaining meals of the day with more calorie-dense options’, ‘When I eat a panini, I start craving more calorie-dense food’, or ‘When I eat pizza for lunch, I will lose control (of my life) and end up in a binge’. It is a challenge to define the feared outcomes in a way that allows for a proper test of the expectation. The therapist and patient should, for example, try to quantify food cravings. The US should be measurable in a way that the patient can investigate and disconfirm the expectation. The monitoring of cravings and intake all day long after eating two croissants, a panini or pizza may help to test the expectations. After many of these exposures, Suzanna concluded: ‘I can have a portion of fries and continue with my day as planned, without ending up in a binge’ or ‘If I have a more calorie-dense meal, it does not make me crave and eat more calorie-dense foods for the rest of the day’.

### 6.2. Fear of Being Judged for Food and Eating

Suzanna was very afraid of being judged by others for her food choices when she was the only person eating in a group. We designed exposure exercises during which she ate different types and amounts of food (variability) alone or in the presence of other people while she observed their reactions. In several exposure sessions, Suzanna ate alone, either in front of the therapist or strangers, who were invited for that particular purpose. In between, she repeated these exposures at home with her family and friends (different contexts). Healthy foods as well as forbidden, more calorie-dense foods were used as CSs. One of her expectations was, for instance, ‘When I am the only person in a group who eats a piece of chocolate, the others will judge me and think I am a glutton’. In addition to eating in front of others, Suzanna was also uncertain about the portions of foods she could eat in the presence of others. She felt it was a bad thing to determine the portion herself, and then felt guilty and afraid of being judged. Therefore, along with using various food stimuli as CSs, self-determining how much to eat was also considered a CS whose association with negative judgement was challenged during exposure sessions. Since ‘being judged’ is a rather vague concept that is difficult to examine, we defined concrete and objective markers for the presence or absence of the expected outcome. Ways to assess judgement are, for example, asking the other person about their thoughts, opinions and feelings in the specific situation. If a patient, however, is skeptical about the credibility of others and assumes that they would respond in a socially desirable manner, asking others to express their thoughts might not be the best way to test the patients’ expectation. The patient might also narrowly observe whether the other person changes their facial expression (eyes or mouth) or gestures in a ‘judgmental’ way. What realistically and objectively characterizes a judgmental facial expression must be carefully determined prior to the actual exposure to combat the risk that patients will interpret the other person’s facial expression in a negatively biased manner. In Suzanna’s case, she defined judgement in two ways. First, she expected other people to make comments on her such as ‘you are weak’, ‘you are fat’, ‘you eat too much’. She thought that people would either directly confront her with such statements or would give this as an answer once she asked them. Second, she defined judgement by facial expressions. She thought that others would move their eyebrows, nose and mouth in a disgusted way. Lastly, the other people invited to the session should receive as little information as possible beforehand to create a more realistic setting and elicit authentic reactions. From these exposures, Suzanna learned that ‘If I eat pizza in front of others, they don’t even observe me eating it’, ‘If I eat more calorie-dense food in front of others, I am not judged negatively’, ‘If I drink a hot chocolate in front of others, they don’t have a negative opinion of it’ or ‘Others don’t judge how much I eat’.

### 6.3. Fear of Being Judged for Weight (Gain) and Appearance

Because of what she had experienced at the sports club, Suzanna developed an intense fear of being weighed and judged by others. She expected, for instance, ‘If others see my weight on the scale they will think I am fat’ or ‘If others see my weight they will think I am weak’. Exposures using the scale offer a broad variety of options to challenge these specific fears. Suzanna, for instance, was weighed in front of the therapist but also in front of strangers who were invited to the session. During these exposures, the other person saw her weight on the scale. Sometimes Suzanna read her weight aloud, as this was even more difficult for her. The scale we used in Suzanna’s treatment could be manipulated, so that confrontations with higher weights could be practiced. Using a manipulable scale means that the weight shown on the scale display is adjusted by a specific application made in our lab, which enables increasing or decreasing one’s body weight by grams or kilograms or by a percentage of the actual weight. The scale display shows the manipulated weight instead of the actual weight, which can be a good exercise to challenge all kinds of feared expectations associated with the higher weight. Even when it is mentioned that the visible weight has been manipulated, standing on the scale can provoke intense anxiety. Like many other patients with anorexia nervosa, the weight Suzanna said she could just barely tolerate and would never want to exceed was far too low, because she feared being judged negatively at a higher weight. Expectations tested with the manipulated scale were, for example, ‘When I weigh 55 kg, the other person will judge me as fat’ or ‘If I weigh 10 kg more and read my weight out loud, others will reject me’. Using the manipulable scale, we added a few kilograms to her actual weight without informing the third person. This allowed Suzanna to observe how the third person reacted to her (increased) weight which she spoke out loud. Of course, the artificially increased weight must be credible to the other person involved. From these exposures, Suzanna learned, for instance, ‘Nothing bad happens when others see my weight’ or ‘Even when my weight is higher, others don’t judge me for it’.

To combat the fear of being judged for body weight and appearance, many patients with anorexia nervosa not only avoid the scale but also tend to dress in a specific way that covers certain body parts. A patient, for instance, might expect that wearing a tighter shirt will result in other people judging her, such as comments about how ‘fat’ she is. Thus, wearing tight clothes becomes a CS and the judgement a US (e.g., ‘when I wear these clothes, my friends will see that I am fat and think I am weak’). The expectation can be challenged in exposure sessions in which the patient dresses differently and tests among friends whether she will actually be judged for it. Again, objective criteria for assessing the expected judgements should be narrowly determined prior to the actual exposure.

After several CS-US expectations related to food, eating and weight were challenged with Suzanna, deepened extinction was applied, during which previously separate CSs are investigated together. Exposures were performed in which she ate forbidden food in front of others, while being weighed before and after eating. In other sessions, she had to decide for herself how much she was going to eat and then consumed the food in front of others, while she was weighed before and after eating, and so on. Expectations that were tested were, for instance, ‘If I eat a piece of chocolate in front of others while wearing heavy clothes and being weighed before and after, they will judge me as a heavy glutton’ or ‘If I eat a self-determined portion of chips in front of others, I will directly gain 400 g and they will think I am a glutton’. After testing such expectations, Suzanna concluded: ‘Others don’t judge me as a glutton’ or ‘Others have no (negative) opinions about my food choices and weight’.

### 6.4. Fear of Emotions Related to Food, Eating, Weight (Gain) and Appearance

In addition to food, eating and weight gain, patients often fear not being able to tolerate the associated emotions. Common expectations include the anxiety of being emotionally overwhelmed and feeling intolerably weak, worthless, distressed, depressed, out of control, guilty or disgusted after eating calorie-dense, forbidden or unfamiliar foods. Fear formulations are, for instance, ‘When I eat a piece of cake, the eating disorder takes over and I cannot tolerate the distress without compensating’ (e.g., by purging, skipping meals, counting calories, exercising), ‘When I drink a coke, my day is ruined, because I cannot stop worrying about it’ or ‘When I eat a piece of pizza, I cannot tolerate how disgusted I am by myself’. Possible exposure exercises to challenge such fears are complex, since it must be defined beforehand what intolerability means and how it can be assessed. The practitioner and patient search for objective markers that allow for the examination of the expectation. Referring to the example about being disgusted after drinking coke, the therapist and patient quantify in advance the level of disgust that is expected to be intolerable (e.g., >7 on a 0–10 scale). In the exposure session, the patient rates the experienced disgust before, during and after drinking the coke to test whether the disgust is as high and as intolerable as expected. Fears of the intolerability of emotions occur not only in the context of food and eating, but also in relation to weight (gain). Suzanna was not only afraid that others would judge her for her weight; she also feared that standing on the scale and being confronted with her weight or actual weight gain would lead to intolerable feelings of distress or loss of control. The (manipulable) scale provides good opportunities to challenge expectations about the intolerability of emotions related to weight (gain). For example, the expectation of intolerable emotions was tested by confronting the patient with the dreaded higher weight on a manipulated scale. One way to challenge the fear of losing control over food intake when confronted with one’s weight is to surprise patients with scale exposures, thereby eliminating the possibility of using safety behaviors in advance (such as extreme food restriction, weighing beforehand or wearing extra-light clothes). Again, when designing the exposure exercises, it is first determined in detail how the expected intolerability of the respective emotion is assessed.

Fears of emotions related to one’s appearance when confronted with one’s own body are also common. Exposure sessions involving a mirror are a suitable option to challenge expectancies about the intolerability of emotions related to appearance. During mirror exposures, the patient stands in front of the mirror and is asked to focus on a disliked body part and to tolerate the emotions that arise without applying safety behaviors [87]. The session is guided by the therapist, who stimulates the patient to continue focusing on the disliked body part. Common expectations challenged during mirror exposures are, for example, ‘I cannot tolerate the disgust I feel when seeing my legs’ or ‘I cannot resist the urge to restrict eating the rest of the day after I see my belly in the mirror’. Sessions in front of the mirror last as long as needed to test and violate the fear expectancy.

Removing safety behaviors that impede inhibitory learning during exposure sessions is necessary, and the therapist and patient must be aware of this. The therapist continuously reminds the patient to refrain from these behaviors, encourages the patient to remain focused on the CS and stimulates the verbalization of feelings during the exposure. Before and after each exposure, the expectations and anxiety are measured, and at the end of the exposure session, it is discussed what was learned. In the beginning, Suzanna did not always manage to refrain from her common safety behaviors. However, the repeated disconfirmation of her expectancies made her less anxious and more confident about food, eating and weight, and it felt easier for her to stop using safety behaviors. Suzanna’s fears, concerns, and eating disorder psychopathology decreased during the exposure therapy (see Table 1) and after treatment, she wrote us a letter describing her experiences (see Box 1).

**Table 1:** Anxiety and eating disorder psychopathology before and after exposure therapy (*n* = 1).

Eating Disorder Psychopathology (EDE-Q)	Eating Concerns (Scale 0–6)	Weight Concerns (Scale 0–6)	Shape Concerns (Scale 0–6)	Eating Restraint (Scale 0–6)	Global Score (Scale 0–6)
Before	4.4	4.4	2.6	1.6	3.3
After	1.4	2.0	1.6	1.0	1.5
Eating Disorder Fear Questionnaire (EFQ)	Fear of weight gain (scale 1–7)	Fear of social consequences (scale 1–7)	Fear of personal consequences (scale 1–7)	Fear of physical sensations (scale 1–7)	Fear of social eating (scale 1–7)
Before	6.5	3.8	5.0	5.7	5.5
After	4	3.5	3.0	3	2
	General anxiety (DASS): normal (0–7), mild (8–9), moderate (10–14), severe (15–19), extremely severe (20+)	Eating anxiety (FOFM) (scale 8–56)	Feared concerns (FOFM) (scale 9–63)	Food avoidance behaviors (FOFM) (scale 8–56)	
Before	22	48	41	19	
After	2	23	21	11	

Anxiety levels and eating disorder psychopathology of Suzanna before and after exposure therapy. Higher scores mean more anxiety, avoidance behaviors and more severe eating disorder psychopathology (Fear of Food Measure (FOFM, [14]; Eating Disorder Fear Questionnaire (EFQ, [15]; Depression Anxiety Stress Scale (DASS, [88,89]; Eating Disorder Examination Questionnaire [90]).

Box 1 **Suzanna’s experiences with the exposure treatment.**

  ‘When I started with the therapy, I was enormously afraid of eating and weigh-ing. Eating a biscuit in front of someone or being weighed felt like the end of the world to me. By then I could already eat again, but the fear remained, and was so big that it determined my whole day. Eating was a daily struggle. Fun things were ru-ined because of the struggle over food, and concentrating on my studies or a good conversation was hard, because my head could only think about food. Whenever I ate, I was always afraid that other people would think I was a glutton and that I would gain weight right away. I felt weak, worthless, and enormously guilty with every bite. Eating felt like an urge I had to suppress, and my eating disorder felt like my lifesaver.

  When I had to do the first scale exposure, I was shaking and rated the fear as 100. Everything inside me was screaming that I should not do it. With the therapists help, I got on it anyway and by acknowledging my feelings at that moment and not pushing them away, not carrying out my subtle safety behaviors and by looking closely at the others’ faces, my fear slowly faded away. Because this time I did not look away, suppress my feelings or quickly step away from the scale, I saw what I had been blind to all those years before: I did not see judgment on their faces, they did not think I was weak, and they did not get angry with me because of my weight. I realized that I am no longer the little, powerless girl from before, in front of adults who force me to do things I do not want to do. I am safe now. I had to go through my fear without performing safety behaviors to see that.

  I ate several biscuits in front of people, I ate fries for lunch and drank chocolate milk with whipped cream for the first time in 10 years. I was not allowed to perform my subtle safety behaviors: I was not allowed to eat very slowly, talk a lot to distract people or look away. Every time I needed these behaviors to cope with my fears confirmed that it was right to be afraid. I looked closely at the therapists’ faces during the eating exposures and assessed whether I was really gaining weight. I looked at what really happened: they didn't disapprove me and I didn’t gain weight immediately. There was actually no evidence at all that confirmed my eating disorder thoughts. In my studies, I would reject a hypothesis if the evidence pointed in the opposite direction. So why hold on to my eating disorder thoughts any longer? Even when other people were around or when we varied exposures—what I feared did not happen. In fact, nothing was happening at all: I was just eating something while other people were around, it was just fries or a number on the scale.

  In exposure therapy, I confronted my fears. I did not just perform a behavior, but I learned to look closely at what was really happening. Do other people think I am weak? Do I really see disapproval on their faces or is that just my own thought? Am I really gaining weight from that one biscuit and is sauce really as fattening as I think? Somehow, I knew my thoughts about food were wrong, but my feelings did not go away with that. I needed to see it before I could believe it, but my avoidance and subtle safety behaviors made me blind for this. In exposure therapy, I learned to open my eyes and really saw that what I am afraid of and what my eating disorder is telling me, is not happening. Through exposure therapy, I learned that my eating disorder is not telling the truth, that you do not gain weight from eating chips once and I learned that eating is normal. I have learned that when the fear comes up, I can handle it.

  My eating disorder is not completely gone, but through exposure therapy, there is now also a second voice in my head. A voice that tells me, when I am scared by my negative eating disorder thoughts, that eating is normal. A voice that tells me that I can eat and that what I am afraid of is not going to happen. There is now a voice in my head pushing down the intensity of my eating disorder. A voice that was not trustworthy to me before this therapy. Through exposure therapy, I can now trust this voice and it has changed how I feel immensely. Before this, I could also imagine that eating was normal and that you do not gain weight from everything, but now I really feel that way. I have also really seen it. Most importantly, I can now just con-centrate on my studies again and I can just engage with my life, friends and family. I am no longer spending all day thinking about how to deal with eating and being in conflict with my eating disorder. Food and I may not yet be best friends, but we are also no longer opposed. I am no longer shocked when my friends spontaneously suggest going for ice cream or to a cafe; then I just feel like doing that with my friends. I can now even suggest eating fries myself, for example. My days have be-come so much nicer and more relaxed now and I feel so much more connected to my life.’

## 7. Challenges and Future Directions

Exposure therapy is a promising intervention to treat eating disorder-related fears, but several challenges need further consideration and more clinical research. Sometimes, patients have difficulties determining what exactly they are afraid of. When it is not possible to formulate concrete hypotheses, however, it is difficult to disconfirm threat expectancies. This is problematic, since the extent to which expectancies change throughout the treatment predicts treatment outcomes [64]. One option is to use exposures to find out what the patients’ feared outcome is. A patient who knows that drinking coke, for instance, will cause anxiety but cannot say why, could be asked to drink the coke and then eventually figure out what the threat expectancy is while actually consuming the drink. Furthermore, some patients detach from their emotions when faced with stressful situations. In consequence, they no longer feel anxiety when they formulate a threat expectancy or even during the actual exposure session. In addition, patients often tend to rationalize, e.g., ‘rationally I know that I cannot gain 1 kg from a piece of pizza’, which, in turn, hinders the process of identifying and disconfirming the fear-driven CS-US association. Generally, it is a delicate balance between conducting an exposure that maximizes the mismatch between expectation and outcome and not ‘losing’ the patient by asking too much. Since patients with anorexia nervosa usually have ambivalent feelings about their treatment, they might be prone to quit faster. When the therapist, however, is too lenient due to the fear of losing the patient, the exposure sessions will remain below their potential. 

In case a patient is too afraid to eat (more calorie-dense) foods at the beginning, using virtual reality (VR) might be a suitable intermediate step. This technology offers the possibility to eat virtually and get in touch with the feared (food) stimulus, while being in a safe surrounding [91]. When eating virtually, controllers are used to hold the virtual cutlery and guide the food to the mouth. Since VR is found to elicit similar emotional reactions to the same situations in real life [92,93], virtual eating offers the opportunity to challenge food-related threat expectancies (e.g., ‘when I eat a piece of cake, I cannot stop worrying about it’). This is advantageous in situations where the patient, for the time being, refuses to eat the food in reality. When fear expectations about specific foods are violated in VR, this may reduce the fear of actually consuming the feared food later on. When challenging threat expectancies related to body, shape and weight, virtual avatars with varying weights that match the patients’ appearance can be used, for example while standing in front of a mirror or in any other environment [94]. VR also offers the opportunity to challenge fears that are otherwise difficult to assess within the therapeutic setting (e.g. visiting a swimming pool). In sum, using VR expands the possibilities in terms of stimuli and settings.

Patients sometimes ‘prepare’ for exposures by applying compensatory behaviors prior to the sessions. Others adjust the remaining meals in the day depending on the exposure content. Especially when testing expectancies about eating and weight gain over several days, it is difficult to determine the effect if the patient adjusts meals and intake in the interim. In addition to rediscussing the aim of exposure sessions (we want to find out what will really happen, so we need to perform the exercise properly), the likelihood of patients using preparatory measures before the exposure sessions can be reduced by surprising the patient with unexpected exposure exercises. Motivation and compliance are continuously worked on by carrying out the exposure exercises and the violation of feared outcomes. Generally, more clinical experience, research and exposure guidelines (such as [58,59]) are needed to find solutions for these challenges in order to achieve the best possible exposure therapy outcomes.

## 8. Conclusions

Exposure therapy is a promising intervention in the treatment of fears associated with anorexia nervosa. This practical guide was intended to share examples and experiences of exposure therapy for anorexia nervosa based on inhibitory learning. Many challenges remain, and much remains to be learned to make exposure interventions as effective as possible; additional clinical research is urgently needed.

## Data Availability

The datasets used and/or analyzed during the current study are available from the corresponding author upon reasonable request.

## Appendix A. Treatment Rationale

In the treatment of anxiety, exposure in vivo is often used. People are exposed to what they are afraid of and what they prefer to avoid. It is important that people discover whether their feared expectations will actually happen. Situations people are afraid of can be both internal, for example, physical sensations, and external, for example, giving a presentation.

Let us take an example. A child falls from her bike and hurts herself badly. She would rather not ride her bicycle again (avoidance) because she is afraid of falling and hurting herself again (expectation). You could tell the child that it is better not to ride the bicycle again or you could encourage the child to get back on the bicycle and tell her that you will help her practice (exposure). By getting back on the bike, the child can learn that she does not always fall when she rides and that it becomes less scary the more she does it.

We know from research that people with eating disorders are often anxious. The fear is often related to eating. An important fear is the fear of gaining weight, and sometimes the fear is to lose control after eating a little (expectation). Because of your fears, you avoid situations such as eating tasty foods, and you do things to feel safe. For example, you try to prevent weight gain by exercising or purging, or you avoid certain situations to prevent you from having to eat again. We call these behaviors safety or avoidance behaviors. They provide relief but they also keep your fear alive. You do not learn whether what you are afraid of will actually happen.

What we will do during this therapy is test whether your fearful expectations will actually come true. We will first find out what you are afraid of. We will have a closer look at your expectations that are associated with these fears. Then, we will investigate whether what you fear will actually happen. We do this by looking for the situations that you are afraid of without avoiding them or using safety behaviors. It is important that the exercises are feasible and that you practice repeatedly in (different) situations. In this way, you acquire new information about situations you are afraid of. You can compare it to motorways in your brain. The fearful and avoidant road is the one that you drive on very quickly and easily; it is an automatic road. With exposure exercises, we try to build a new road. In the beginning, you will notice that it is easier to drive on the familiar automatic road. The more you drive on the new road, the more it will become the new highway you like to drive on. This means that in the beginning, you have to actively choose the new highway, but eventually, it will become the new automatic road.

This therapy requires a lot of courage, because we seek out the situations that are very fearful for you, and we try to not use the safety behaviors that are so familiar for you. Your fears may be strong in the beginning. That is normal, but you will also note that the intensity will change. I am sure you can do it and, of course, I will help you do the right things.

After the treatment, you will be able to decide for yourself what, when and where to eat instead of letting the eating disorder determine this and limit your freedom.

## Appendix B. Exposure Plan

Before the exposure session

What is my expectation?	If………………………………………… Then……………………………………….
What do you do to prevent this from happening? What do you not do to prevent this from happening?	
How can we examine whether your expectation comes true?	

During the exposure session

What do you feel right now? Do you in a certain way hold back the fear or the things that you are afraid of?	

Post-discussion

What did really happen? Are you surprised by the result? If yes, why?	
In case your expectation did not decrease: - Do you have an idea why? - Is it possible that you did things to reduce your anxiety? Did you do something to prevent your expectation coming true?	
What did you learn from this exposure? What is your conclusion?	

Follow-up

Were you anxious enough during the exposure? Can you make it more difficult for yourself? Can you combine this exposure?	

## Appendix C. Visual Analogue Scale for Threat Expectancies and Anxiety

Indicate with a vertical line (|) what you are currently feeling:

Indicate with a vertical line (|) what you are currently feeling:
